# Shorter Time to Biopsy of Patients with Head and Neck Squamous Cell Carcinoma During the COVID-19 Pandemic in Hungary

**DOI:** 10.3390/cancers17030360

**Published:** 2025-01-23

**Authors:** Éva Szabó, Eszter Kopjár, László Rumi, Szabolcs Bellyei, Antal Zemplényi, Emese Mátyus, Eszter Édes, János Girán, István Kiss, István Szanyi, Éva Pozsgai

**Affiliations:** 1 Department of Otorhinolaryngology, University of Pécs Clinical Center, Munkácsy M. Street 2, 7621 Pécs, Hungary; 2 Urology Clinic, University of Pécs Clinical Center, Munkácsy Mihaly Street 2, 7621 Pécs, Hungary; 3 Department of Oncotherapy, University of Pécs Clinical Center, Édesanyák Street 17, 7624 Pécs, Hungary; 4 Center for Health Technology Assessment and Pharmacoeconomics Research, University of Pécs Faculty of Pharmacy, Rákóczi Street 2, 7623 Pécs, Hungary; 5 Department of Public Health Medicine, University of Pécs Medical School, Szigeti Street 12, 7624 Pécs, Hungary; 6 Department of Primary Health Care, University of Pécs Medical School, Rákóczi Street 2, 7623 Pécs, Hungary

**Keywords:** head and neck squamous cell carcinoma, COVID-19, waiting time, delay, time to treatment, time to biopsy

## Abstract

Background/objectives: The goal of this investigation was to compare the time to biopsy (TBI) and time to treatment (TTI) for head and neck squamous cell carcinoma (HNSCC) patients before and during the COVID-19 pandemic and to examine the effect of demographic and clinical characteristics on these intervals. Methods: Our retrospective study at a large regional Hungarian cancer center analyzed data from patients aged 18 or older diagnosed with HNSCC between 1 January 2017 and 15 March 2020 (pre-COVID-19 period) and 16 March 2020 to 13 May 2021 (COVID-19 period). We calculated the time from initial physician contact to biopsy (TBI) and from biopsy to treatment initiation (TTI) and performed descriptive and exploratory statistical analyses. Results: The median TBI decreased significantly (6 vs. 3 days; *p* = 0.008), while the median TTI was not affected significantly (28 vs. 29 days; *p* = 0.972) pre-pandemic and during the pandemic, respectively. Residence in a village was linked to a significant reduction in median TBI during the pandemic (*p* = 0.000), coinciding with a higher proportion of rural patients diagnosed with oral cavity/oropharyngeal cancers during the pandemic (50.3% pre-pandemic vs. 67.4% during pandemic, *p* = 0.044). Median TTI decreased significantly during the pandemic for patients with laryngeal tumors (27.5 vs. 18.5 days; *p* = 0.012). Conclusions: Our study, one of a few from this region, provides insights into HNSCC patient waiting times. Improvement in TBI likely resulted from the availability of telemedicine, reduced diagnostic demands from non-cancer patients, and an increased incidence of oral cavity/oropharyngeal cancer among rural patients.

## Simple Summary

The COVID-19 pandemic profoundly affected cancer care. This study aimed to compare waiting times, specifically time to biopsy (TBI) and time to treatment (TTI), for patients with head and neck squamous cell carcinoma (HNSCC) before and during the pandemic. We retrospectively analyzed data from adult HNSCC patients across two periods: 1 January 2017 to 15 March 2020 (pre-pandemic) and 16 March 2020 to 13 May 2021 (pandemic). The median TBI decreased significantly from 6 to 3 days, while the median TTI remained unchanged between the two periods. Residence in a village was associated with a significant reduction in median TBI during the pandemic, and there was a higher proportion of rural patients diagnosed with oral cavity/oropharyngeal cancers (50.3% pre-pandemic vs. 67.4% during the pandemic). Improved TBI likely resulted from the availability of telemedicine, reduced diagnostic demands from non-cancer patients, and an increased incidence of oral cavity/oropharyngeal cancers among rural patients.

## 1. Introduction

The COVID-19 pandemic has significantly impacted cancer care worldwide. Research has shown that, across many countries, cancer patients experienced delays in care, including extended times to treatment [1,2,3,4]. Patients with certain types of cancer, such as head and neck squamous cell carcinoma (HNSCC), were often diagnosed at more advanced stages, resulting in an increased cancer burden and lower survival rates as a result of the pandemic [3,5]. Notably, the number of new diagnoses for head and neck cancers, particularly among elderly patients, declined sharply [6,7].

HNSCC constitutes the seventh most common cancer globally, accounting for 4.6% of all cancer-related deaths [8,9]. Despite advances in diagnostic and therapeutic methods over recent decades, the global 5-year survival rates for HNSCC have remained largely unchanged over the past 30 years, with annual incidence rates rising [8,9].

According to GLOBOCAN estimates, Hungary has the highest cancer incidence and mortality rates in Europe [8,10], including the highest rates of oral and pharyngeal cancer morbidity and mortality in Europe and the third highest mortality rate globally [11].

Since prolonged waiting times for treatment are associated with a significantly increased risk of local cancer recurrence, reducing delays in diagnosis and treatment for HNSCC has become a crucial goal within healthcare systems. [12,13]

Studies investigating the waiting times of patients with HNSCC in the context of the pandemic have therefore gained increased attention since the onset of the pandemic, yielding mixed results. Based on the results of some studies, there was a significant delay in the treatment of patients with HNSCC [4,14,15] while others reported no notable delays during the pandemic [5]. According to Szewcy et al., both the mean time from tumor board presentation to treatment initiation and from first visit to treatment increased significantly, from 17.1 to 21.7 days and from 44.7 to 54.4 days, respectively [15]. Similarly, a study from London reported significant increases in the time from referral to treatment for HNSCC patients during the pandemic [14]. In contrast, Tevetoglu et al. and Nishimura et al. reported no statistically significant difference between the pre-pandemic and pandemic waiting times [2,4]. In a previous study, we reported a bidirectional shift in cancer stage distribution due to the pandemic and a longer time from appearance of symptoms to initial physician contact [16].

Analyses of HNSCC patient waiting times in Central Europe are limited but essential to understanding the pandemic’s effects on cancer care in different regions.

The primary aim of our present study was to analyze and compare the time to biopsy (TBI) and time to treatment (TTI) intervals for patients with HNSCC before and during the COVID-19 pandemic at a large regional cancer center in Hungary. Additionally, we investigated whether certain demographic and clinical characteristics influenced these healthcare-related waiting times. Finally, we compared the time to death (TTDe) of patients between the pre-pandemic and pandemic periods.

## 2. Methods

### 2.1. Study Setting

Prior to the study, ethical approval was obtained from the Regional Ethical Committee (Reference number: 8850-PTE2021).

The investigation was carried out at a Hungarian regional clinical and cancer center, at the University of Pécs Clinical Center’s Department of Otorhinolaryngology and Head and Neck Surgery (UP ENT) in Pécs, Hungary. The clinic provides care for cancer patients from Baranya County and partially for patients from neighboring Tolna and Somogy counties in the Transdanubian region of Hungary. The clinic includes a specialized cancer center comprising an inpatient unit, a day oncology unit, and a radiotherapy unit.

The following is a description of the functioning of the healthcare pathways before and during the COVID-19 pandemic.

Before the COVID-19 pandemic, patients experiencing symptoms of any disease—including those related to the head and neck—would typically visit their family physician, or in urgent cases, the emergency department. For symptoms specific to the head and neck region, patients also had the option of scheduling an outpatient ENT (or in some cases dentist/oral surgeon) consultation directly, bypassing the need for a referral from a general practitioner (GP) or emergency physician.

During the pandemic, the healthcare system adapted by shifting non-urgent cases—including those related to the head and neck—to telemedicine, typically managed via telephone consultations with family physicians. However, patients seeking appointments with ENT specialists, dentists, or oral surgeons for symptoms suggestive of malignancy were examined and treated in person at the clinic.

Following the Hungarian government’s announcement of a national safety crisis on 16 March 2020, elective surgeries were postponed in healthcare centers across the country, and surgeons as well as dentists were instructed to treat only emergency cases. However, due to the low number of active COVID-19 cases, elective surgeries could resume within certain limits from May to November 2020, though the country remained in a state of pandemic preparedness. Throughout the pandemic, oncological and emergency surgeries, cardiology interventions, and reproduction-related procedures were exempt from these restrictions. All restrictions were lifted on 13 May 2021.

### 2.2. Study Design

This observational, retrospective investigation included patients aged 18 or older who visited the UP ENT and had a histological confirmation of squamous cell carcinoma of mucosa-epithelial origin in the oral cavity, pharynx, and larynx, or cytology-confirmed cervical lymph node metastasis of an unknown primary tumor (CUP). Consequently, patients with an International Classification of Diseases, 10th Revision (ICD-10) code of C00–C06, C09-C14, or C32 were included. Patients were excluded from the study if they had a history of any other tumor within five years prior to their diagnosis of HNSCC or if they had secondary tumors [16].

The investigation was divided into two study periods: the first, the “pre-COVID-19” period, between 1 January 2017 to 15 March 2020, and the second, the “COVID-19” period from 16 March 2020 to 13 May 2021, the latter based on guidelines issued by the Hungarian National Directorate General for Hospitals concerning alterations in healthcare provision during the COVID-19 pandemic (as described above and previously) [16].

The Clinical Center’s electronic database, the eMedSolution Integrated Healthcare IT System, served as the data source for our study. This database contains the electronic medical records of all patients receiving both inpatient and outpatient care at the University of Pécs Clinical Center and is accessible to healthcare professionals across the entire institution. Using automated data extraction methods, the database was screened for patients with the specified ICD-10 diagnosis codes, and demographic data such as age, gender, and place of residence were collected and exported to an Excel file. Clinical data, including tumor stage, localization, and dates used to calculate waiting times (TBI, TTI, TTDe), were manually collected by the research team. [16]. Patients were followed up for 24 months following the initial physician contact and—where applicable—the death of the patients was recorded. Tumor location, presenting symptoms, and diagnoses following admission to UP ENT were categorized according to the ICD-10 [16].

### 2.3. Definition of Waiting Times

Date of initial physician contact was the date of the patient first contacting any physician (general practitioner, dentist/oral surgeon, ENT specialist, or other specialist) with their symptoms.

The time to biopsy interval (TBI) was the number of days between the initial physician contact and the date of the sampling of the tumor (which was performed under local or general anesthesia depending on the localization of the tumor, or in the case of a CUP, fine-needle aspiration biopsy).

The time to treatment interval (TTI) was the number of days between the biopsy of the tumor and the date of the first day of any form of treatment (surgery or oncological treatment) the patient received, as described previously in the literature [17].

The time to death interval (TTDe) was the number of days between the initial physician contact and the death of the patient.

**Figure 1:**
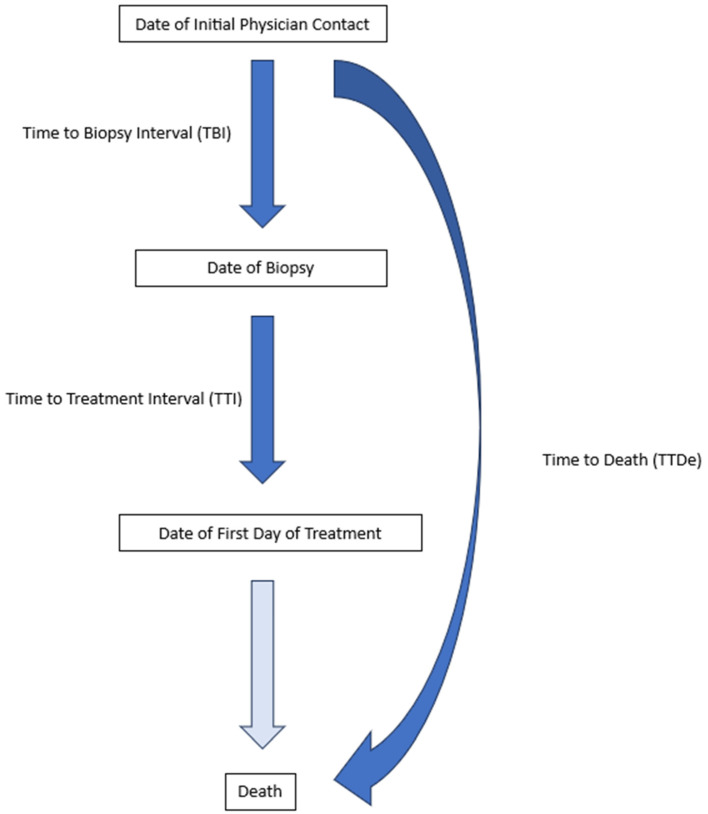
The investigated waiting times of HNSCC patients in our study (shown in dark blue).

Figure 1 shows the investigated time intervals.

The primary outcome measures for this study were the comparison of the median healthcare-related waiting times TBI, TTI as well as the TTDe of HNSCC patients between the pre-COVID-19 and during the COVID-19 periods. The secondary outcome measures were the analyses of demographic and clinical characteristics on the median of TBI and TTI waiting times.

Investigated demographic and clinical factors included the characteristics of HNSCC patients (*n* = 525) visiting the UP ENT in the two, pre-COVID-19 and COVID-19, study periods depicted in Table 1, as also described previously [16].

### 2.4. Data Analysis

To address the study’s research questions, we devised a data analysis framework, then descriptive and exploratory statistical analysis was carried out. Frequency tables were utilized to characterize the demographic, clinical, and treatment profiles of the patients. To examine the stochastic nature of the relationships between the analyzed data, we used the chi-square test, with *p* ≤ 0.05. For the analysis of the median values of TBI, TTI, and TTDe, we employed the Mann–Whitney test. Logistic regression analysis was carried out to analyze the impact of demographic and clinical factors on the healthcare-related waiting times TBI and TTI, as well as the effect of TBI on TTDe. Statistical analyses were conducted using Jamovi 2.2.5.

## 3. Results

### 3.1. Comparison of Healthcare-Related Waiting Times (TBI, TTI) and Time to Death (TTDe) of HNSCC Patients Between the Two Study Periods

**Figure 2:**
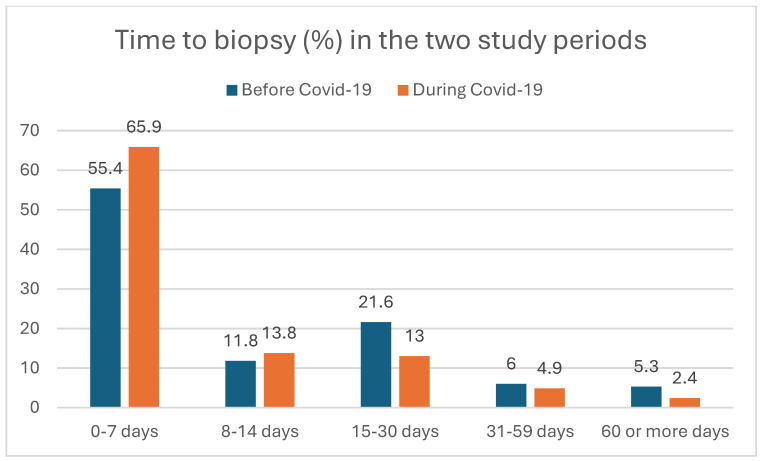
Time from initial physician contact to biopsy (time to biopsy, TBI) of HNSCC patients (in percentages) before and during the COVID-19 period.

**Figure 3:**
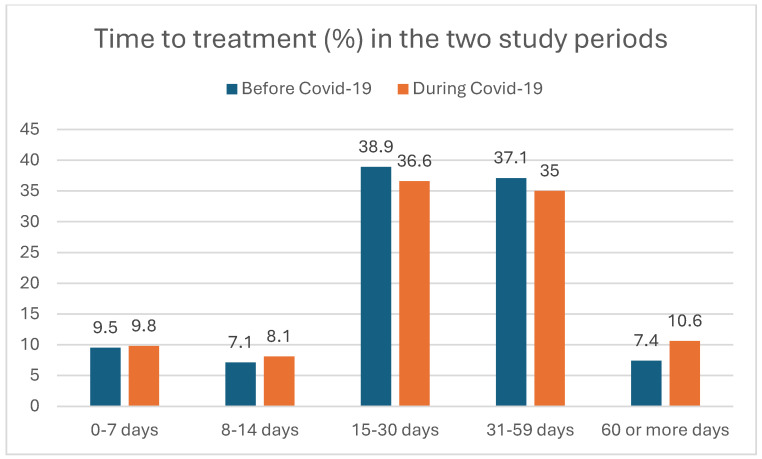
Time from biopsy to the first day of treatment (time to treatment, TTI) of HNSCC patients (in percentages) before and during the COVID-19 period.

**Figure 4:**
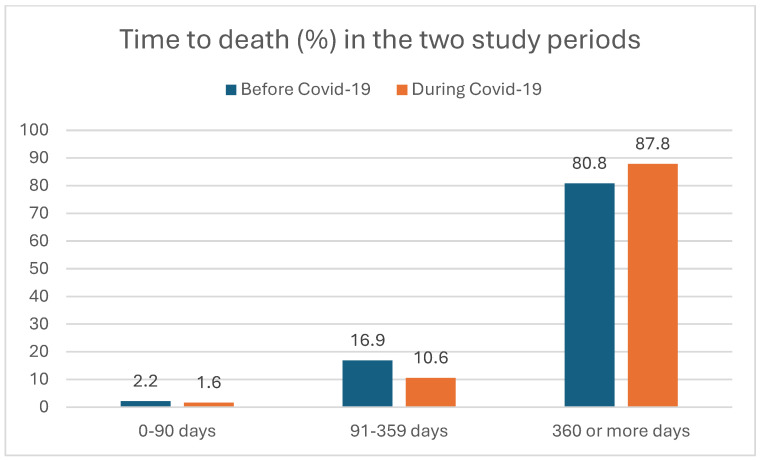
Time between the appearance of initial symptoms to death of the patient (time to death, TTDe) of HNSCC patients (in percentages) before and during the COVID-19 period.

We analyzed the healthcare-related waiting times (TBI, TTI) and time to death (TTDe) of HNSCC patients between the pre-pandemic and pandemic periods. Figure 2, Figure 3 and Figure 4 show the distribution of the TBI, TTI, and TTDe in days, and Table 2 indicates their median values.

The median time to biopsy decreased significantly from 6 to 3 days during the pandemic (*p* = 0.008), as shown in Table 2. The median time to treatment did not show a significant difference between the two study periods, with a TTI of 28 days in the pre-pandemic and 29 days during the pandemic periods (*p* = 0.972) (Table 2).

Although the median TTDe was longer before the pandemic compared to the COVID-19 era, this difference was not significant either (404 vs. 315 days, respectively, *p* = 0.136). (Table 2).

### 3.2. Comparison of the Effect of Demographic and Clinical Factors on TBI and TTI Before and During the Pandemic

When comparing the effect of demographic and clinical factors on TBI in the two study periods, we found that residence in a village or a city (other than the county seat) was associated with significantly decreased median TBIs during the pandemic (from 7 to 0 days; *p* = 0.000, and from 6 to 2 days; *p* = 0.049, respectively). The specialty of the initially contacted physician, if the physician was a dentist/oral surgeon, also showed a significant relationship with the median TBIs before and during the pandemic. Finally, the median TBIs decreased significantly for stage II and stage IVc cancers during the pandemic, from 7 to 0 days; *p* = 0.007, and from 6.5 to 0 days; *p* = 0.019, respectively (Table 3).

However, no significant changes were detected for any other cancer stage (stages I, III, IVa, IVb). Neither gender, tumor localization, the place of residence in the county seat nor if the initially contacted physician was other than a dentist/oral surgeon showed a significant association with the median TBIs. These results are shown in Table S1.

When comparing the effect of demographic and clinical factors on TTI in the two study periods, we found that the median TTI decreased significantly during the pandemic if the patient resided in the county seat (from 32.5 to 21 days; *p* = 0.002) or if the patient’s tumor was localized in the larynx (from 27.0 to 18.5 days; *p* = 0.012) (Table 4). However, neither the gender of the patient, the stage of the patient’s tumor, the tumor localization, the place of residence in a village or a city nor the specialty of the initially contacted physician influenced the median TTIs significantly in the two study periods (Table S2).

Tumor localization may influence the time to biopsy (TBI) and time to treatment (TTI), as patients with more accessible tumor types, such as oral cavity or oropharyngeal cancers, may undergo biopsy on the same day as their initial physician visit. In contrast, biopsies for less accessible cancers, like laryngeal cancer, generally require separate appointments under anesthesia. To explore this, we examined whether a significant relationship existed between oral cavity/oropharyngeal cancers and factors associated with median TBI (Table 3) and median TTI (Table 4).

**Figure 5:**
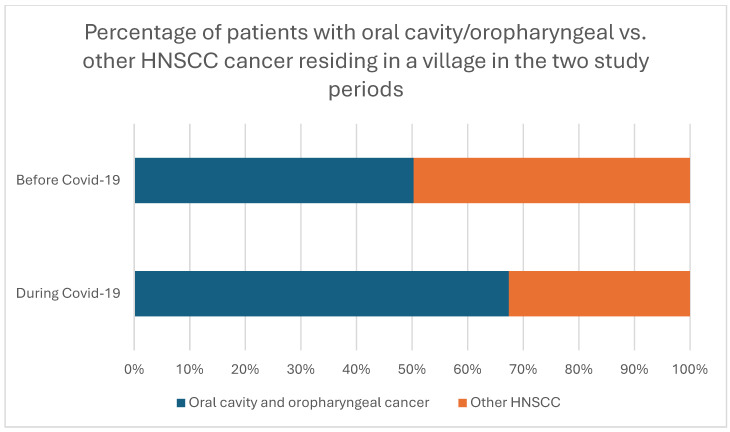
Distribution of patients with oral cavity/oropharyngeal vs. other HNSCC cancer residing in a village in the two study periods (*p* = 0.044).

Our analysis revealed a significant association between rural residency (residency in a village) and oral cavity/oropharyngeal cancer during the COVID-19 pandemic, thus indicating that a significantly higher percentage of rural patients with oral cavity/oropharyngeal cancers were diagnosed during the pandemic compared to before (50.3% pre-pandemic vs. 67.4% during the pandemic, *p* = 0.044; shown in Figure 5). No other significant relationships between the factors in Table 3 and Table 4 and patients with oral cavity/oropharyngeal cancer were identified before or during the pandemic.

## 4. Discussion

The COVID-19 pandemic impacted healthcare systems worldwide, from the heightened burden of caring for infected patients to implementing regulations aimed at influencing patients’ behaviors when seeking medical care. The effect on oncological care has also been studied, as the treatment of cancer, including those with HNSCC, could not be delayed despite the restrictions. Our study focused on the impact of the COVID-19 pandemic on healthcare-related waiting times for patients with HNSCC at a large regional cancer center in Hungary.

A number of studies have analyzed the effect of the pandemic on the characteristics and waiting times of head and neck cancer patients, yielding mixed results. Longer times to diagnosis were found in the US by Yao et al. [18] and in Poland by Kanicka et al., where median times to diagnosis increased from 16 to 20 days [19]. However, an Italian study and an investigation in West Scotland found no significant change in time to diagnosis during the pandemic [20,21], while a nonsignificant decrease in time to diagnosis was recorded in Canada, with a decrease from 14.1 to 9.9 days (*p* = 0.142) [22]. In line with this Canadian study, we also found a decrease in median time to initial diagnostic sampling of the tumor; however, our results proved significant, with a 3-day reduction in waiting time.

Possible underlying reasons for quicker diagnostic testing in our study may include the overall decrease in patient burden during the pandemic, as patients with non-urgent symptoms and illnesses were encouraged to stay at home [4,6]. Additionally, all elective interventions, such as surgeries, were stopped, while time-sensitive care, including urgent and oncological treatments, was prioritized. Furthermore, as other studies have noted, there was a decrease in the overall number of patients diagnosed with HNSCC during the pandemic, ranging from 7.5% to 50% [4,6,17,23,24]. In our study, we observed a nonsignificant decrease of 12.4%, as previously reported [16]. However, other studies have documented no change in patient volume [22]. These changes likely contributed to the availability of human and diagnostic resources being focused on the time-sensitive and smaller number of HNSCC patients in our investigation.

Additional literature has been published on the time to treatment for HNSCC patients. A significant increase in the time to treatment was reported in Germany (45 vs. 35 days, *p* = 0.004) for all HNSCC patients [25] and Croatia (21.5 vs. 31.5 days for laryngeal cancer, *p* = 0.001; and 8.58 vs. 8.155 days for oral cancer, *p* = 0.006) [3]. However, other studies reported no significant change, including Gazzini et al. in Italy (28.9 vs. 30.4 days, *p* = 0.77) [20], Tevetoglu et al. in Turkey [2], Kourtaidis [26], and Zubair et al. [14]. Our findings are consistent with these results, as the median time to treatment for HNSCC patients increased slightly from 28 to 29 days during the pandemic (*p* = 0.972).

Conversely, some recent studies suggested a significant reduction in time to treatment during the pandemic, with decreases from 31–32 days to 26–28 days (*p* < 0.001) in the Netherlands, and from 76.6 to 48.7 days (*p* = 0.0001) in Canada [17,22]. These reductions may be attributed to changes in healthcare system regulations and a decrease in the number of patients presenting to hospitals, as described earlier [4,6].

We investigated whether demographic or clinical factors were associated with TBI during the two study periods. Early-stage (stage II) and very advanced-stage (stage IVc) cancers, rural residence (outside the county seat), and primary contact with a dentist/oral surgeon were significantly associated with decreased TBI during the pandemic. Our results indicate that patients with these characteristics experienced shorter diagnostic waiting times during the pandemic compared to before.

The proportion of patients with advanced-stage cancers has been reported to have increased during the pandemic, according to recent studies [5,12,27]. However, no significant difference was found between the proportions of early- and advanced-stage cancers in our previous analysis [16], consistent with findings from a Polish study [19]. Patients with advanced cancers often require faster and more urgent care, which may explain the significant association of stage IVc cancers with shorter TBI we found in our present study. On the other hand, the significant association of shorter TBI among stage II cancers was possibly due to reduced patient burden and increased diagnostic capacity among healthcare providers. These changes may have resulted from restrictions on non-urgent cases and an overall decrease in patient numbers during the pandemic [16].

Residence in rural areas has been shown to negatively affect patient health by limiting access to healthcare. The pandemic further exacerbated these health inequalities [28] as demonstrated in a Hungarian national study [29]. Contrary to our expectations, residing in a village other than the county seat was associated with significantly shorter TBI. This finding may be explained by the increased incidence of rural patients with oral cavity or oropharyngeal cancer during the pandemic. Biopsies for these cancers are easier to perform, often conducted on the same day as the patient’s visit, unlike biopsies for other head and neck cancers, such as laryngeal cancer, which typically require scheduling and are conducted under general anesthesia. Additionally, shorter TBI was significantly linked to initial consultations with dentists/oral surgeons, and patients with symptoms related to oral cavity cancer often contact a dentist/oral surgeon with their symptoms.

It must be added that other factors, such as comorbidities—which were not analyzed in our study—or altered patient pathways may also have contributed to the shorter TBI of rural patients. Before the pandemic, rural patients had more limited in-person access to primary healthcare physicians compared to those in the county seat. However, during the pandemic, access to primary care became possible via telephone or other telemedicine modalities, possibly reducing this disparity [30].

Conversely, residing in the county seat where the cancer center was located and having a laryngeal tumor were significantly associated with shorter treatment times. These findings may partly be explained by a previous study reporting a higher proportion of HNSCC patients from urban areas than rural ones, as shorter distances to healthcare providers facilitate access to surgery or oncological treatment [19]. The shorter median TTI for laryngeal tumors during the COVID-19 period may be attributed to the findings from our previous article that patients with laryngeal cancer were more likely to visit a physician at an early stage of their disease [16]. For instance, in the case of stage I laryngeal (vocal cord) tumors, direct laryngoscopy often served as both biopsy and treatment, with complete lesion removal reducing the time to treatment. Furthermore, treatment of laryngeal cancer may also have benefited from reduced patient loads and fewer non-urgent, non-oncological surgeries during the pandemic compared to pre-pandemic levels [30,31], although reduced times to treatment were not observed for other HNSCC cancers during the pandemic.

Our study showed a decrease in time to death during the pandemic, though this difference was not significant. Since the cause of death was not reliably recorded, we can only hypothesize that the shorter time to death may be due to various factors. These include death from COVID-19, to which cancer patients are more susceptible [32,33], or a decline in general health caused by reduced attention to non-urgent medical conditions, such as chronic diseases [34]. 

The distribution of treatment modalities was likewise not significantly influenced by the pandemic. These findings align with those of Psycharis et al., who also reported that treatment algorithms were unaffected by the pandemic [22]. This suggests that some cancer centers were not significantly impacted by guideline modifications introduced during the pandemic.

## 5. Limitations

Certain limitations of this study might affect the interpretation of our findings. This was a single-center study and may not represent national trends. Secondly, the comparison of median waiting times for TBI and TTI between countries may be limited by variations in patient pathways and regulations between countries during the pandemic.

## 6. Conclusions

Nearly five years after its onset, researchers continue to strive to understand and analyze the effects of the COVID-19 pandemic. Understanding its short-term and long-term effects on patient pathways is crucial for improving care for this vulnerable group of cancer patients, whose diagnosis and treatment must be prioritized, even during emergencies like the pandemic. Exploring regional and national differences provides a broader perspective on how patient pathways changed during the pandemic and their impact on patient waiting times and delays.

Our study found that the median time to biopsy decreased significantly during the pandemic. Certain cancer stages, the patient’s place of residence, and the specialty of the initially contacted physician were significantly associated with this reduction. An increase in the percentage of oral cavity and oropharyngeal cancer among rural patients was also observed. Notably, no significant differences were found in the initial therapeutic regimens between the two study periods nor in the median times to treatment and death.

Factors such as the widespread availability of telemedicine, reduced diagnostic demands from non-cancer patients, an increase in the incidence of oral cavity or oropharyngeal cancer among rural patients, the prioritization of cancer patients due to the time sensitivity of their treatment [35], and fewer HNSCC cases overall [16,22] possibly contributed to the positive impact on TBI observed in our study. However, it is important to note that these encouraging findings may have come at the cost of untreated non-cancer conditions and the potential underdiagnosis or oversight of non-urgent cancer cases.

Nonetheless, our study is one of the few from the Central-Eastern European region and provides valuable insight into the patient pathways of HNSCC patients during a healthcare crisis, such as the pandemic.

## Figures and Tables

**Table 1:** The demographic (A), clinical (B), and initial treatment characteristics (C) of HNSCC patients visiting the UP ENT before and during the COVID-19 period [16].

(A)				
	Total *n* = 525 (%)	Before COVID-19 *n* = 402 (%)	During COVID-19 *n* = 123 (%)	*p*
**Sex**				0.434
**Male**	427 (*81.3*)	324 (*80.6*)	103 (*83.7*)	
**Female**	98 (*18.7*)	78 (*19.4*)	20 (*16.3*)	
**Age groups (years)**				0.708
**18–54**	78 (*14.8*)	59 (*14.7*)	19 (*15.4*)	
**55–59**	88 (*16.7*)	68 (*16.9*)	20 (*16.3*)	
**60–64**	107 (*20.4*)	82 (*20.4*)	25 (*20.3*)	
**65–69**	117 (*22.3*)	88 (*21.9*)	29 (*23.6*)	
**70–74**	63 (*12*)	45 (*11.2*)	18 (*14.6*)	
**75<**	72 (*13.7*)	60 (*14.9*)	12 (*9.8*)	
**Place of residence**				0.203
**County seat**	140 (*26.7*)	109 (*27.1*)	31 (*25.2*)	
**Other city**	175 (*33.3*)	126 (*31.3*)	49 (*39.8*)	
**Village**	210 (*40*)	167 (*41.5*)	43 (*35*)	
**(B)**				
	**Total** ***n* = 525 (%)**	**Before COVID-19** ***n* = 402 (%)**	**During COVID-19** ***n* = 123 (%)**	***p***
**Tumor site**				0.507
**Lips and oral cavity**	152 (*28.9*)	112 (*27.9*)	40 (*32.5*)	
**Oropharynx**	137 (*26.1*)	105 (*26.1*)	32 (*26*)	
**Hypopharynx**	93 (*17.8*)	70 (*17.4*)	23 (*18.7*)	
**Larynx**	120 (*22.9*)	94 (*23.4*)	26 (*21.1*)	
**Epipharynx**	8 (*1.5*)	8 (*2*)	0 (*0*)	
**CUP (carcinoma of unknown primary)**	15 (*2.9*)	13 (*3.2*)	2 (*1.6*)	
**Specialty of initially contacted physician**				0.441
**ENT specialist**	384 (*73.4*)	291 (*72.4*)	93 (*75.6*)	
**Dentist**	112 (*21.3*)	86 (*21.4*)	26 (*21.1*)	
**Other**	29 (*5.5*)	25 (*6.2*)	4 (*3.3*)	
**Tumor stage**				0.199
**Early-stage (I-II)**	155 (*29.5*)	113 (*28.1*)	42 (*34.1*)	
**Late-stage (III-IV)**	370 (*70.5*)	289 (*71.9*)	81 (*65.9*)	
**(C)**				
	**Total**	**Before COVID-19**	**During COVID-19**	***p***
	***n* = 518 (%)**	***n* = 395 (%)**	***n* = 123 (%)**	
**Surgery**	174 (*33.6*)	134 (*33.9*)	40 (*32.5*)	0.278
**Chemoirradiation**	132 (*25.5*)	95 (*24.1*)	37 (*30.1*)	
**Radiotherapy**	119 (*23.0*)	87 (*22.0*)	32 (*26.0*)	
**Induction Chemotherapy**	63 (*12.2*)	53 (*13.4*)	10 (*8.1*)	
**Chemotherapy**	16 (*3.1*)	13 (*3.2*)	3 (*2.4*)	
**Basic supportive care**	14 (*2.7*)	13 (*3.2*)	1 (*0.8*)	

**Table 2:** Median TBI, TTI, and TTDe of HNSCC patients before and during the COVID-19 period.

	Before COVID-19	During COVID-19	*p*
**Median Time to Biopsy (TBI)** (days)	6.00	3.00	0.008
**Median Time to Treatment (TTI)** (days)	28.00	29.00	0.972
**Median Time to Death (TTDe)** (days)			
	404	315	0.136

**Table 3:** Demographic and clinical factors showing a significant association with median TBI in the two study periods among patients with HNSCC.

Time to Biopsy (TBI) (Median Days)	Before COVID-19	During COVID-19	*p*
**Cancer stage**			
Stage II	7.00	0.00	0.007
Stage IVc	6.50	0.00	0.019
**Place of residence**			
Village	7.00	0.00	0.000
Other City	6.00	2.00	0.049
**Specialty of initially contacted physician**			
Dentist/Oral surgeon	6.00	0.00	0.009

**Table 4:** Demographic and clinical factors showing a significant association with median TTI in the two study periods among patients with HNSCC.

Time to Treatment (TTI) (Median Days)	Before COVID-19	During COVID-19	*p*
**Place of residence**			
County seat	32.5	21.00	0.002
**Tumor site**			
Larynx	27.00	18.50	0.012

## Data Availability

The datasets used and/or analyzed during the current study are available from the corresponding author on reasonable request.

## Supplementary Materials

The following supporting information can be downloaded at: https://www.mdpi.com/article/10.3390/cancers17030360/s1, Supplementary Table S1. Demographic and clinical factors of HNSCC patients showing no significant association with TBI in the two study periods; Supplementary Table S2. Demographic and clinical factors of HNSCC patients showing no significant association with TTI in the two study periods.

## Abbreviations

CUP	Carcinoma of unknown primary.
ENT	Otolaryngologist.
HNSCC	Head and neck squamous cell carcinoma.
ICD-10	International Classification of Diseases, tenth revision.
TBI	Time to biopsy interval.
TTI	Time to treatment interval.
TTDe	Time to death interval.
UP CC ENT	University of Pécs Clinical Centre, Department of Otorhinolaryngology and Head and Neck Surgery.

## References

[1] Kościelecka K.E., Kuć A.J., Kubik D.M., Męcik-Kronenberg T., Ceglarz D. (2021). Impact of the COVID-19 Pandemic on the Availability of Medical Care Among Oncological Patients. Wiad. Lek..

[2] Tevetoğlu F., Kara S., Aliyeva C., Yıldırım R., Yener H.M. (2021). Delayed presentation of head and neck cancer patients during COVID-19 pandemic. Eur. Arch. Otorhinolaryngol..

[3] Gršić K., Blivajs I., Pastorčić Grgić M., Prgomet D., Lukinović J., Vugrinec O., Matoc L., Miličić B., Leović D. (2022). The Impact of COVID-19 on Head and Neck Cancer Treatment Delay. Acta Clin. Croat..

[4] Nishimura N.Y., Aoki K., Koyama S., Nishio M., Otsuka T., Miyazaki M., Yoshii T., Otozai S., Miyabe J., Korematsu M. (2024). The impact of COVID-19 pandemic on head and neck cancer diagnosis and treatment. J. Dent. Sci..

[5] Mettias B., Charlton A., Ashokkumar S. (2021). Outcome of two-week head and neck cancer pathway for the otolaryngology department in a tertiary centre. J. Laryngol. Otol..

[6] Solis R.N., Mehrzad M., Faiq S., Frusciante R.P., Sekhon H.K., Abouyared M., Bewley A.F., Farwell D.G., Birkeland A.C. (2021). The Impact of COVID-19 on Head and Neck Cancer Treatment: Before and During the Pandemic. OTO Open.

[7] Peacock H.M., De Gendt C., Silversmit G., Nuyts S., Casselman J., Machiels J.P., Giusti F., van Gool B., Vander Poorten V., Van Eycken L. (2023). Stage shift and relative survival for head and neck cancer during the 2020 COVID-19 pandemic: A population-based study of temporal trends. Front. Oncol..

[8] Sung H., Ferlay J., Siegel R.L., Laversanne M., Soerjomataram I., Jemal A., Bray F. (2021). Global Cancer Statistics 2020: GLOBOCAN Estimates of Incidence and Mortality Worldwide for 36 Cancers in 185 Countries. CA Cancer J. Clin..

[9] Gormley M., Creaney G., Schache A., Ingarfield K., Conway D.I. (2022). Reviewing the epidemiology of head and neck cancer: Definitions, trends and risk factors. Br. Dent. J..

[10] Bencina G., Chami N., Hughes R., Weston G., Golusiński P.J. (2022). Lost productivity due to head and neck cancer mortality in Hungary, Poland, and Romania. J. Cancer Policy.

[11] Dyba T., Randi G., Bray F., Martos C., Giusti F., Nicholson N., Gavin A., Flego M., Neamtiu L., Dimitrova N. (2021). The European cancer burden in 2020: Incidence and mortality estimates for 40 countries and 25 major cancers. Eur. J. Cancer.

[12] Webb C.J., Benton J., Tandon S., Jones T.M., Roland N.J. (2007). Head and neck cancer waiting times. Clin. Otolaryngol..

[13] Lyhne N.M., Christensen A., Alanin M.C., Bruun M.T., Jung T.H., Bruhn M.A., Jespersen J.B., Kristensen C.A., Andersen E., Godballe C. (2013). Waiting times for diagnosis and treatment of head and neck cancer in Denmark in 2010 compared to 1992 and 2002. Eur. J. Cancer.

[14] Zubair A., Jamshaid S., Scholfield D.W., Hariri A.A., Ahmed J., Ghufoor K., Ali S. (2023). Impact of COVID-19 pandemic on head-neck cancer referral and treatment pathway in North East London. Ann. R. Coll. Surg. Engl..

[15] Szewczyk M., Pazdrowski J., Golusiński P., Pazdrowski P., Więckowska B., Golusiński W. (2021). The impact of the COVID-19 pandemic on the management of head and neck cancer patients at a tertiary care institution in Poland. Contemp. Oncol..

[16] Szabó É., Kopjár E., Rumi L., Boronkai Á., Bellyei S., Gyöngyi Z., Zemplényi A., Sütő B., Girán J., Kiss I. (2024). Changes in Time to Initial Physician Contact and Cancer Stage Distribution during the COVID-19 Pandemic in Patients with Head and Neck Squamous Cell Carcinoma at a Large Hungarian Cancer Center. Cancers.

[17] Schoonbeek R.C., de Jel D.V.C., van Dijk B.A.C., Willems S.M., Bloemena E., Hoebers F.J.P., van Meerten E., Verbist B.M., Smeele L.E., Halmos G.B. (2022). Fewer head and neck cancer diagnoses and faster treatment initiation during COVID-19 in 2020: A nationwide population-based analysis. Radiother. Oncol..

[18] Yao P., Cooley V., Kuhel W., Tassler A., Banuchi V., Long S., Savenkov O., Kutler D.I. (2021). Times to Diagnosis, Staging, and Treatment of Head and Neck Cancer Before and During COVID-19. OTO Open.

[19] Kanicka M., Chabowski M., Rutkowska M. (2023). A Single-Center Study of the Impact of the COVID-19 Pandemic on the Organization of Healthcare Service Delivery to Patients with Head and Neck Cancer. Cancers.

[20] Gazzini L., Fazio E., Dallari V., Accorona R., Abousiam M., Nebiaj A., Giorgetti G., Girolami I., Vittadello F., Magnato R. (2022). Impact of the COVID-19 pandemic on head and neck cancer diagnosis: Data from a single referral center, South Tyrol, northern Italy. Eur. Arch. Otorhinolaryngol..

[21] Drake I., Rogers A., Stewart M., Montgomery J. (2022). The impact of coronavirus disease 2019 on the head and neck cancer pathway in the West of Scotland. J. Laryngol. Otol..

[22] Psycharis S.S., Salameh S., Turkdogan S., Razzaq S., Zhao K., Mascarella M.A., Richardson K., Mlynarek A.M., Hier M.P., Sadeghi N. (2023). Prioritization of head and neck cancer patient care during the COVID-19 pandemic: A retrospective cohort study. J. Otolaryngol. Head Neck Surg..

[23] Rygalski C.J., Zhao S., Eskander A., Zhan K.Y., Mroz E.A., Brock G., Silverman D.A., Blakaj D., Bonomi M.R., Carrau R.L. (2021). Time to Surgery and Survival in Head and Neck Cancer. Ann. Surg. Oncol..

[24] Decker K.M., Feely A., Bucher O., Czaykowski P., Hebbard P., Kim J.O., Pitz M., Singh H., Thiessen M., Lambert P. (2023). New Cancer Diagnoses Before and During the COVID-19 Pandemic. JAMA Netw. Open.

[25] Metzger K., Mrosek J., Zittel S., Pilz M., Held T., Adeberg S., Ristow O., Hoffmann J., Engel M., Freudlsperger C. (2021). Treatment delay and tumor size in patients with oral cancer during the first year of the COVID-19 pandemic. Head Neck.

[26] Kourtidis S., Münst J., Hofmann V.M. (2022). Effects of the COVID-19 Pandemic on Head and Neck Cancer Stage and Treatment Duration. Cureus.

[27] Clements K., Thapa A., Cowell A., Conway D., Douglas C.M., Paterson C. (2024). Impact of the COVID-19 pandemic on stage and incidence of head and neck cancer: A rapid review and meta-analysis. Clin. Otolaryngol..

[28] Markey C., Bello O., Hanley M., Loehrer A.P. (2023). The Use of Area-Level Socioeconomic Indices in Evaluating Cancer Care Delivery: A Scoping Review. Ann. Surg. Oncol..

[29] Bekele B.B., Alhaffar B.A., Wasnik R.N., Sándor J. (2022). The Effect of the COVID-19 Pandemic on the Social Inequalities of Health Care Use in Hungary: A Nationally Representative Cross-Sectional Study. Int. J. Environ. Res. Public Health.

[30] Bezerra G.M.F., de Lucena Feitosa E.S., Vale Catunda J.G., Nogueira Sales Graça C., Lucena de Aquino P., Bezerra Neto A.G., Bezerra da Silva Junior G. (2022). Telemedicine Application and Assessment During the COVID-19 Pandemic. Stud. Health Technol. Inform..

[31] Karamani L., McLean A.L., Kamp M.A., Mayer T.E., Müller W., Dinc N., Senft C. (2023). Tumor size, treatment patterns, and survival in neuro-oncology patients before and during the COVID-19 pandemic. Neurosurg. Rev..

[32] Yang K., Sheng Y., Huang C., Jin Y., Xiong N., Jiang K., Lu H., Liu J., Yang J., Dong Y. (2020). Clinical characteristics, outcomes, and risk factors for mortality in patients with cancer and COVID-19 in Hubei, China: A multicentre, retrospective, cohort study. Lancet Oncol..

[33] Liang W., Guan W., Chen R., Wang W., Li J., Xu K., Li C., Ai Q., Lu W., Liang H. (2020). Cancer patients in SARS-CoV-2 infection: A nationwide analysis in China. Lancet Oncol..

[34] Rabbone I., Schiaffini R., Cherubini V., Maffeis C., Scaramuzza A. (2020). Has COVID-19 Delayed the Diagnosis and Worsened the Presentation of Type 1 Diabetes in Children?. Diabetes Care.

[35] Khorana A.A., Tullio K., Elson P., Pennell N.A., Grobmyer S.R., Kalady M.F., Raymond D., Abraham J., Klein E.A., Walsh R.M. (2019). Time to initial cancer treatment in the United States and association with survival over time: An observational study. PLoS ONE.

