# Supplementary material for: Shorter Time to Biopsy of Patients with Head and Neck Squamous Cell Carcinoma During the COVID-19 Pandemic in Hungary
Source: Cancers (Basel). 2025 Jan 23;17(3):360. doi: 10.3390/cancers17030360 (PMC11815749; doi:10.3390/cancers17030360)
Supplement: Supplementary file 1 [file cancers-17-00360-s001.zip › Supplementary Table S1.pdf]

**Supplementary Table S1** Demographic and clinical factors of HNSCC patients showing no significant association with TBI in the two study periods

| | Before Covid-19 | During Covid-19 | p |
|---|---|---|---|
| **Time to Biopsy (TBI)** (median days) | | | |
| **Tumor stage** | | | |
| I stage | 14.00 | 4.00 | 0.165 |
| III stage | 7.00 | 9.00 | 0.735 |
| IVa stage | 3.00 | 5.00 | 0.914 |
| IVb stage | 10.5 | 5.5 | 0.13 |
| **Sex** | | | |
| female | 7 | 5.5 | 0.685 |
| **Place of residence** | | | |
| County seat | 4.5 | 9.00 | 0.125 |
| **Specialty of initially contacted physician** | | | |
| ENT specialist | 6.00 | 5.00 | 0.134 |
| Other | 10.00 | 3.5 | 0.374 |
| **Tumor site** | | | |
| oropharynx | 1.00 | 0.00 | 0.272 |
| hypopharynx | 10.00 | 8.00 | 0.321 |
| larynx | 15.00 | 8.00 | 0.188 |
| CUP (carcinoma of unknown primary) | 7.00 | 11.00 | 0.933 |
