# Supplementary material for: Shorter Time to Biopsy of Patients with Head and Neck Squamous Cell Carcinoma During the COVID-19 Pandemic in Hungary
Source: Cancers (Basel). 2025 Jan 23;17(3):360. doi: 10.3390/cancers17030360 (PMC11815749; doi:10.3390/cancers17030360)
Supplement: Supplementary file 1 [file cancers-17-00360-s001.zip › Supplementary Table S2.pdf]

Supplementary Table 2. Demographic and clinical factors of HNSCC patients showing no significant association with TTI in the two study periods

	**Before Covid-19**	**During Covid-19**	**p**
**Time to Treatment (TTI)** (median days)			
**Tumor stage**			
I stage	21	28	0.246
II stage	27.5	31.00	0.540
III stage	29.5	25.00	0.248
IVa stage	30.00	31.00	0.691
IVb stage	29.00	26.5	0.934
IVc stage	26.00	16.00	0.208
**Sex**			
female	30.5	29.5	0.919
male	28.00	29.00	0.997
**Place of residence**			
Other city	27.00	30.00	0.095
Village	28.00	31.00	0.313
**Specialty of initially contacted physician**			
ENT specialist	41.00	34.00	0.300
Dentist	36.00	51.00	0.052
Other	42.00	34.5	0.924
**Tumor site**			
oral cavity	27.00	31.00	0.164
oropharynx	31.00	31.5	0.561
hypopharynx	26.5	28.00	0.799
CUP (cancer of unknown primary)	62.5	61.00	1.000
